# Excitatory and inhibitory contributions to local field potentials in human and monkey

**DOI:** 10.1186/1471-2202-14-S1-P107

**Published:** 2013-07-08

**Authors:** Bartosz Teleńczuk, Alain Destexhe

**Affiliations:** 1 Unité de Neurosciences, Information & Complexité, Centre National de la Recherche Scientifique, 91198 Gif-sur-Yvette, France

## 

Local field potentials (LFPs) are a common measure of the neuronal population activity. However, their origins are not yet fully understood. A recent in vitro study has shown that even single spikes emitted by an interneuron can trigger detectable unitary field potentials in extracellular medium [1]. Their contributions can be additionally amplified due to strong spatial correlations in interneuronal firing [2]. Nevertheless, inhibitory activity in vivo is strongly correlated with the excitatory potentials leading to effective cancellations of generated field potentials. As a result, the unitary field potentials induced by interneuronal spikes can not be separated from background activity. Here, we describe a technique that allows to disambiguate the excitatory and inhibitory contributions.

We study the contribution of excitatory and inhibitory neurons in awake and sleeping brain of human and monkey. The experimental data were recorded intracortically in patients accepted for the surgical treatment of epileptic foci. We discriminated 82 single units over a electrode grid of 92 electrodes (covering an area of 4 mm × 4 mm) together with accompanying local field potentials. We compared the results with LPFs and spikes recorded from electrode arrays of the same geometry implanted in motor cortex of macaque monkeys [3]. For both datasets, we determined the field potential components related to spikes of inhibitory neurons by means of spike-triggered average (STA) and covariance (STC) methods [4].

First, we determined the spatial reach of the spike-induced field by means of spike-triggered average analysis. We found that the potentials triggered by putative interneurons propagate across the electrode grid at velocity higher than the field of putative pyramidal neurons (propagation velocity: 0.5 m/s and 0.3 m/s, respectively). In addition, the spike-triggered covariance analysis revealed gamma-band components in the local field potentials of interneurons but not pyramidal neurons. These high-frequency components are most likely related to the oscillations generated within a local network. To test this hypothesis, we simulate a network of simplified neurons that manifest gamma-band synchronization. Next, we apply the spike-triggered analysis to the field potentials calculated as a local average of the membrane potentials. The results of the simulations are compared to the experimental data.

We demonstrate that spike triggered covariance allows to separate components that are not accessible to standard techniques due to cancellations of the field produced by inhibitory and excitatory potentials. The application of this technique to the activities recorded in interneurons revealed previously unidentified components of local field potentials. Computer simulations suggest that these components are related to nonlinear interactions within the networks of inhibitory and excitatory neurons. Interestingly, we have not found such gamma-band components in the LFPs associated with excitatory neurons. Overall, our results point to essentially different relations between LFPs and spikes of excitatory and inhibitory neurons. We propose that this difference may provide a new electrophysiological criterion for the identification of interneurons.
